# Rhamnolipid Nano-Micelles versus Alcohol-Based Hand Sanitizer: A Comparative Study for Antibacterial Activity against Hospital-Acquired Infections and Toxicity Concerns

**DOI:** 10.3390/antibiotics11050605

**Published:** 2022-04-29

**Authors:** Yasmin Abo-zeid, Marwa Reda Bakkar, Gehad E. Elkhouly, Nermeen R. Raya, Dalia Zaafar

**Affiliations:** 1 Department of Pharmaceutics and Industrial Pharmacy, Faculty of Pharmacy, Helwan University, Cairo 11795, Egypt; gehad.elkhouly@pharm.helwan.edu.eg (G.E.E.); nermeen.rayh@pharm.helwan.edu.eg (N.R.R.); 2 Helwan Nanotechnology Center, Helwan University, Cairo 11792, Egypt; 3 Botany and Microbiology Department, Faculty of Science, Helwan University, Cairo 11795, Egypt; marwa_mahmoud01@science.helwan.edu.eg; 4 Pharmacology and Toxicology Department, Modern University for Technology and Information, Cairo 12055, Egypt; dalia.zaffar@pharm.mti.edu.eg

**Keywords:** multi-drug resistant bacteria, rhamnolipids nano-micelles, ethyl-alcohol, hospital-acquired infections

## Abstract

Hospital-acquired infections (HAIs) are considered to be a major global healthcare challenge, in large part because of the development of microbial resistance to currently approved antimicrobial drugs. HAIs are frequently preventable through infection prevention and control measures, with hand hygiene as a key activity. Improving hand hygiene was reported to reduce the transmission of healthcare-associated pathogens and HAIs. Alcohol-based hand sanitizers are commonly used due to their rapid action and broad spectrum of microbicidal activity, offering protection against bacteria and viruses. However, their frequent administration has been reported to be associated with many side effects, such as skin sensitivity, skin drying, and cracks, which promote further skin infections. Thus, there is an essential need to find alternative approaches to hand sanitation. Rhamnolipids are glycolipids produced by *Pseudomonas aeruginosa*, and were shown to have broad antimicrobial activity as biosurfactants. We have previously demonstrated the antimicrobial activity of rhamnolipid nano-micelles against selected drug-resistant Gram-negative (*Salmonella* Montevideo and *Salmonella* Typhimurium) and Gram-positive bacteria (*Staphylococcus aureus*, *Streptococcus pneumoniae*). To the best of our knowledge, the antimicrobial activity of rhamnolipid nano-micelles in comparison to alcohol-based hand sanitizers against microorganisms commonly causing HAIs in Egypt—such as *Acinetobacter baumannii* and *Staphylococcus aureus*—has not yet been studied. In the present work, a comparative study of the antibacterial activity of rhamnolipid nano-micelles versus alcohol-based hand sanitizers was performed, and their safety profiles were also assessed. It was demonstrated that rhamnolipid nano-micelles had a comparable antibacterial activity to alcohol-based hand sanitizer, with a better safety profile, i.e., rhamnolipid nano-micelles are unlikely to cause any harmful effects on the skin. Thus, rhamnolipid nano-micelles could be recommended to replace alcohol-based hand sanitizers; however, they must still be tested by healthcare workers in healthcare settings to ascertain their antimicrobial activity and safety.

## 1. Introduction

Hospital-acquired infections (HAIs) are considered to be a major source of morbidity and mortality, and are the second most prevalent cause of death globally [1]. The World Health Organization (WHO) estimate that out of every 100 hospitalized patients at any given time, 7 in developed and 10 in developing countries—including Egypt—will acquire at least one HAI [2]. The risk of acquiring HAIs is significantly higher in intensive care units (ICUs), with approximately 30% of patients having at least one infection [2]. HAIs can lead to a prolonged hospital stay, long-term disability, increased antimicrobial resistance, additional financial burden for healthcare systems, high costs for patients and their families, and excess deaths [2]. In the Middle East region, the literature reports various HAI rates caused by a wide range of microorganisms, with various patterns of antimicrobial resistance [3,4,5,6,7,8,9,10,11].

Healthcare workers’ hands are considered a standard vehicle for the transmission and spread of healthcare-associated pathogens from one patient to another and within the healthcare environment [12,13]. Thus, maintaining hand hygiene is considered the key activity to control/prevent HAIs [12]. This has been further confirmed by the ongoing COVID-19 outbreak; given the seriousness of this outbreak, it can be observed that hand hygiene now occupies a new place of importance in the minds of healthcare workers, as well as among people in the community [14]. Compliance with hand hygiene by healthcare workers alone during the COVID-19 pandemic was reported to be effective in reducing HAIs—including the transmission of SARS-CoV-2—in hospital settings [12,14,15]. Hand washing with soap and water or hand rubbing with alcohol-based hand sanitizers are commonly adopted methodologies for hand hygiene [16,17].

Alcohol-based hand sanitizers are commonly used due to their rapid action and broad spectrum of microbicidal activity, offering protection against bacteria and viruses [1]. However, their frequent application as revealed by the ongoing COVID-19 pandemic has been reported to be associated with several hazards to humans and the environment [14,18]. For example, irritation and allergic conditions of the skin with prolonged exposure can result in dryness or cracking of the skin, along with peeling redness or itching [15] and the possibility of skin damage, hence reducing its ability to work as a barrier against other harmful pathogens, and this has been reported to increase the possibility of further infections by microorganisms [19]. Overuse of alcohol-based hand sanitizers in some cases has also been reported to increase the risk of viral outbreaks [19,20], such as increased risk of norovirus [21], as well as the development of antimicrobial resistance [19,22,23]. Therefore, there is a high demand to find novel alternative strategies for hand sanitation with minimal adverse effects.

Rhamnolipids (Rhas) are biosurfactants that are abundantly produced by *Pseudomonas aeruginosa* [24,25]. Rhamnolipids have been reported to be used in the cosmetics and medicines industries [26]. A cost-effective large-scale manufacture of rhamnolipids has been established [27]. This involved a long journey to find cheap renewable substrates [28,29], optimize fermentation conditions [30], reduce the cost of downstreaming [31,32], and produce a genetically engineered strain of *P. aeruginosa* to magnify the quantity of rhamnolipids produced to meet the requirements of industry [33]. Evonik is a stock-listed German specialty chemicals company headquartered in Essen, North Rhine-Westphalia, Germany, and reported a massive investigation of the large-scale production of rhamnolipids for commercial applications [27,34], as well as building the world’s first industrial-scale rhamnolipid production plant [35].

We previously reported the antimicrobial activity of rhamnolipid nano-micelles against selected multidrug-resistant bacteria and SARS-CoV-2 [14], and recommended them to replace alcohol-based hand sanitizers. However, to the best of our knowledge, the antimicrobial activity of rhamnolipid nano-micelles in comparison to alcoholic-based hand sanitizers has not yet been studied. Therefore, the present study constitutes the first assessment of the antimicrobial activity and safety of rhamnolipid nano-micelles compared to a commonly used alcohol-based hand sanitizer (ethyl alcohol 70%). Two pharmaceutical hand sanitizer formulations (solution and gel) of rhamnolipid nano-micelles and ethyl alcohol 70% were prepared in our lab, and their antibacterial activity was investigated against multidrug-resistant bacteria that are commonly known to cause HAIs in Egypt: *Acinetobacter baumannii*, and *Staphylococcus aureus* [36]. The cytotoxicity of these formulations to human dermal fibroblast cells was also assessed to ascertain the viability of rhamnolipid nano-micelles to replace alcohol-based hand sanitizers in hospitals [37].

## 2. Results

### 2.1. Production of Rha(s)

The successful production of rhamnolipids was confirmed using an ESI-MS spectrometer coupled with UPLC (LC/ESI-MS), with the data agreeing with the literature [14]. The obtained rhamnolipids were composed of a higher proportion of mono-rhamnolipids to di-rhamnolipids (Table S1, Supplementary Materials).

### 2.2. Preparation and Characterization of Antimicrobial Formulations

Rhamnolipid nano-micelle formulations were prepared as described in Section 4.2.2 and Section 4.2.3. The particle size and zeta potential were recorded using a Malvern Zeta-sizer instrument, and are presented in Table 1 as average diameter (D, nm) ± SD and average zeta potential (mv) ± SD, respectively. As revealed, the particle size and zeta potential of the nano-micelles solution and gel prepared at 10 mg/mL were 129 ± 4.14 nm, −67.97 ± 2.56 mv and 263 ± 19.13 nm, −38.03 ± 9.24 mv, respectively. The polydispersity index (PDI) values for the solution and gel formulations prepared at 10 mg/mL indicated a monodisperse sample (PDI ≤ 0.3). Dilution of the samples was accompanied by an increase in particle size in solution for all diluted samples compared to undiluted samples, unlike the gel formulation, where a large particle size was recorded initially, but further dilution reduced the particle size. Dilution of samples of both formulations was also accompanied by an increase in PDI values (PDI > 0.3). The values of zeta potential recorded for both formulations were above −30, indicating highly stable samples. However, rhamnolipid nano-micelles in gel showed a lower zeta potential value than solution for the same concentration of nano-micelles.

**Figure 1:**
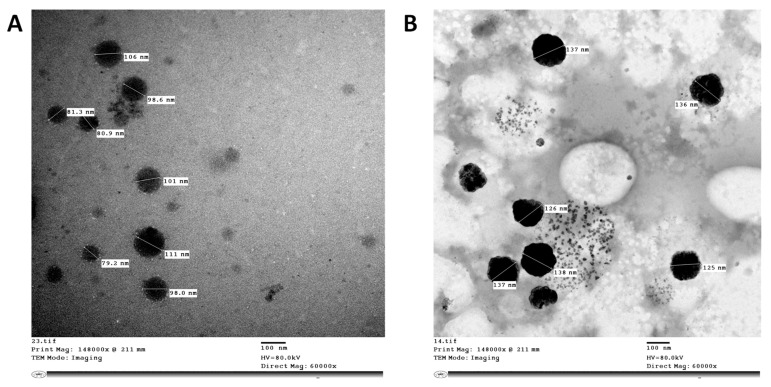
TEM images of rhamnolipid nano-micelles in (**A**) solution and (**B**) gel prepared at a concentration 10 mg/mL of rhamnolipids.

Transmission electron microscopy images of rhamnolipid nano-micelles solution and gel formulations prepared at a concentration of 10 mg/mL are presented in Figure 1. The images show spherical nano-micelles with no sign of aggregation, and the particle size ranges from 79 to 106 nm and from 125 to 138 nm for the solution and gel, respectively. The particle size was smaller than that recorded with the Malvern Zeta-sizer instrument.

The pH and gel viscosity for rhamnolipid nano-micelles and ethyl alcohol 70% formulations were determined as described in Section 4.2.4. The viscosity was equal to 2094 ± 19 and 2957 ± 22 cps for the rhamnolipid nano-micelles and ethyl alcohol 70% gels, respectively. The viscosity (2344 ± 25 cps) of the commercial alcohol-based gel hand sanitizer was less than the viscosity of the ethyl alcohol 70% gel, and slightly higher than the viscosity recorded for the rhamnolipid nano-micelles gel prepared in our lab; however, our prepared formulations showed a good consistency to be applied on the skin.

The pH measured for the rhamnolipid nano-micelles solution and gel were 6.32 ± 0.02 and 5.9 ± 0.2, respectively, while the pH values for the ethyl alcohol 70% solution and gel were 6.2 ± 0.19 and 5.88 ± 0.03, respectively. These values are close to the pH of the commercial hand sanitizer solution (6 ± 0.2) and gel (5.88 ± 0.02).

### 2.3. Determination of Antibacterial Activity

**Figure 2:**
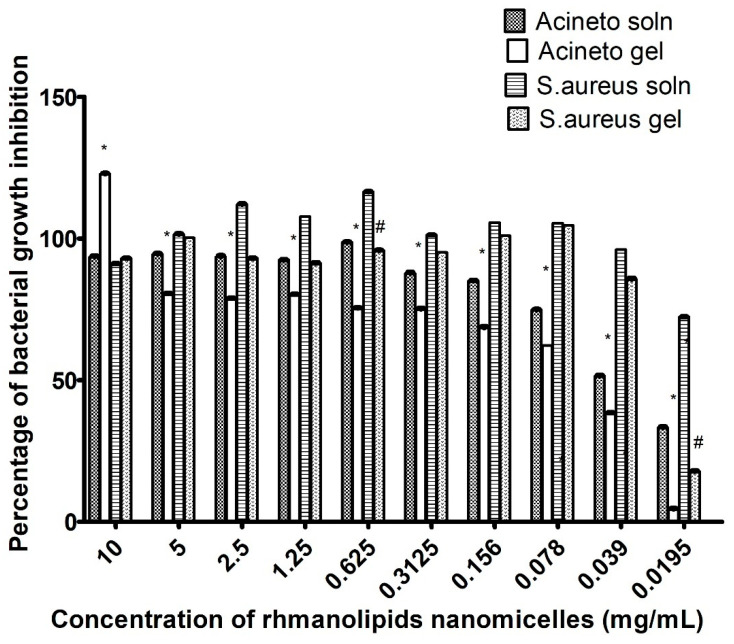
Percentage of bacterial growth inhibition of *A. baumannii* and *S. aureus* recorded by microdilution assay at different concentrations of rhamnolipid nano-micelles formulations (solution and gel). Data are expressed as the mean ± SD. Data were analyzed by two-way ANOVA followed by Bonferroni’s test for multiple comparisons. Significant differences in the antibacterial potency of the rhamnolipid nano-micelles formulations (solution and gel) against *A. baumannii* and *S. aureus* are denoted by * and # at *p* < 0.05, respectively.

The prepared formulations of rhamnolipid nano-micelles and ethyl alcohol 70% were investigated for their antibacterial activity against *S. aureus* and *A. baumannii*. The ethyl alcohol 70 % formulations (solution and gel) inhibited the growth of both bacteria completely (100% inhibition of bacterial growth). The antibacterial activity of the rhamnolipid nano-micelle formulations is presented in Figure 2. For *S. aureus,* the rhamnolipid nano-micelle solution showed a percentage of bacterial growth inhibition ≥ 100% at nano-micelle concentrations ranging from 0.039 to 5 mg/mL. However, at nano-micelle concentrations of 0.0195 and 10 mg/mL, a lower percentage of bacterial inhibition was recorded, at 70 and 96%, respectively.

The rhamnolipid nano-micelle gel inhibited the growth of *S. aureus* completely (≥100%) at nano-micelle concentrations of 0.078, 0.156, and 5 mg/mL, while a lower percentage of bacterial growth inhibition was recorded with other nano-micelle concentrations.

By comparing the antibacterial activity of the rhamnolipid nano-micelle solution and gel against *S. aureus*, it was found that the solution significantly (*p* < 0.05) inhibited the growth of *S. aureus* to a higher extent than the gel formulation at nano-micelle concentrations of 0.0195 and 0.625 mg/mL.

In comparison to *A. baumannii,* the rhamnolipid nano-micelle solution had a percentage of bacterial growth inhibition ranging from 51% to 100% over a nano-micelles concentration range of 0.039 to 10 mg/mL. At a lower concentration of nano-micelles (0.0195 mg/mL), a lower percentage of bacterial growth inhibition (33%) was recorded. The rhamnolipid nano-micelles solution significantly (*p* < 0.05) inhibited the growth of *A. baumannii* to a greater extent than the nano-micelles gel over all concentrations except 10 mg/mL.

The MICs recorded for the rhamnolipid nano-micelles solution against *S. aureus* and *A. baumannii* were 0.039 mg/mL and 0.312 mg/mL, respectively. Conversely, significantly (*p* < 0.05) higher MIC values were recorded with gel formulations: 0.078 mg/mL and 0.625 mg/mL for *S. aureus* and *A. baumannii*, respectively. As revealed from the MIC values, we can conclude that the solution had a significantly (*p* < 0.05) greater potency against both bacteria than the gel formulation. Thus, time–kill curve studies and TEM were performed only for the solution of rhamnolipid nano-micelles.

**Figure 3:**
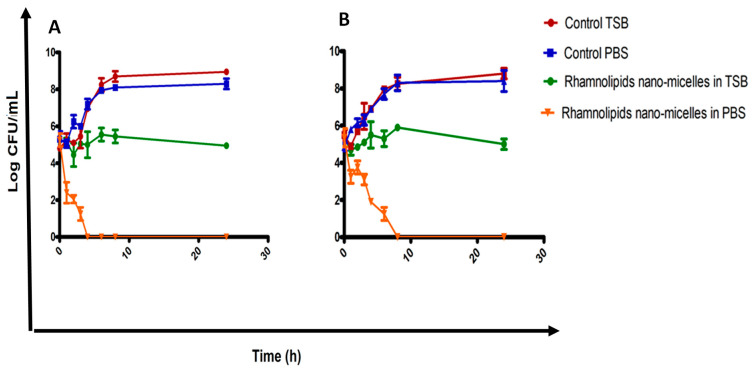
Time–kill curve of antibacterial activity of the rhamnolipid nano-micelles solution at its MIC value against (**A**) *S. aureus* and (**B**) *A. baumannii*; each time point is the average of two independent experiments with three replicates each.

The time–kill curves for the rhamnolipid nano-micelle solution against *S. aureus* and *A. baumannii* are presented in Figure 3A,B, respectively. As revealed, for both bacteria, changing the medium from TSB to PBS to run the time–kill curve assay was not accompanied by any significant (*p* > 0.05) effect on the bacterial count (Log cfu/mL). However, the growth of bacteria in control TSB and PBS (zero concentration of rhamnolipid nano-micelles) increased from 5 Log cfu/mL (initial inoculum of bacteria) to around 9 log cfu/ mL for both bacteria after incubation for 24 h. By tracking the bacteria-killing rate in TSB and PBS at the MIC of rhamnolipid nano-micelles, a significant (*p* < 0.05) reduction in bacterial count (Log cfu/mL) was observed in PBS for both bacteria compared to control PBS. The latter indicates the killing of bacteria due to the presence of rhamnolipid nano-micelles, and the complete eradication of bacteria was detected only after 4 and 8 h for *S. aureus* and *A. baumannii*, respectively. Conversely, with TSB, although the incubation was performed in the presence of the MIC of rhamnolipid nano-micelles, the initial bacterial inoculum was maintained at 5 log cfu/mL for both bacteria.

**Figure 4:**
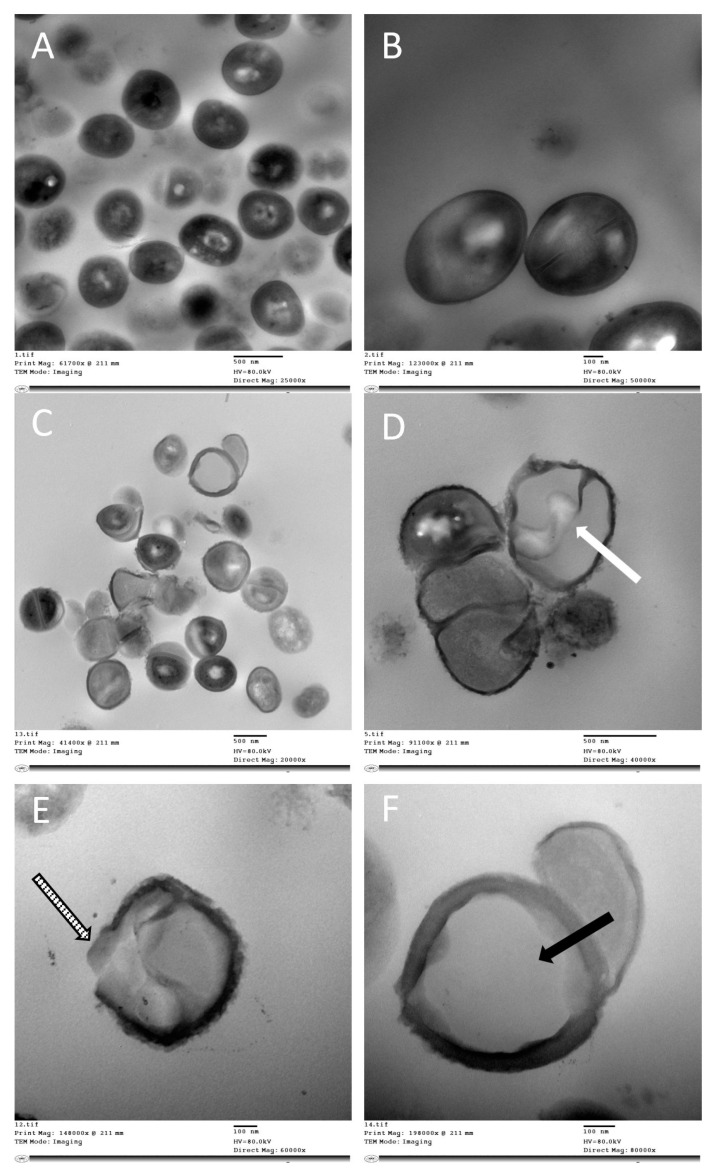
TEM images presenting the structural changes in *Staphylococcus aureus* after treatment with rhamnolipid nano-micelles at concentrations less than the MIC. Images (**A**,**B**) show a normal cell wall outline without any itching or degradation, and with a normal central plane of division and complete cellular components. Image (**C**) shows abnormal cell morphologies. Images (**D**–**F**) show the possible antibacterial mechanisms recorded for the rhamnolipid nano-micelle solution: (**D**) abnormal cell division as revealed by an incomplete central plane of division (white arrow); (**E**) abnormal, degraded, itched, and permeabilized cell wall (striped arrow); and (**F**) bleaching of intracellular components (black arrow).

**Figure 5:**
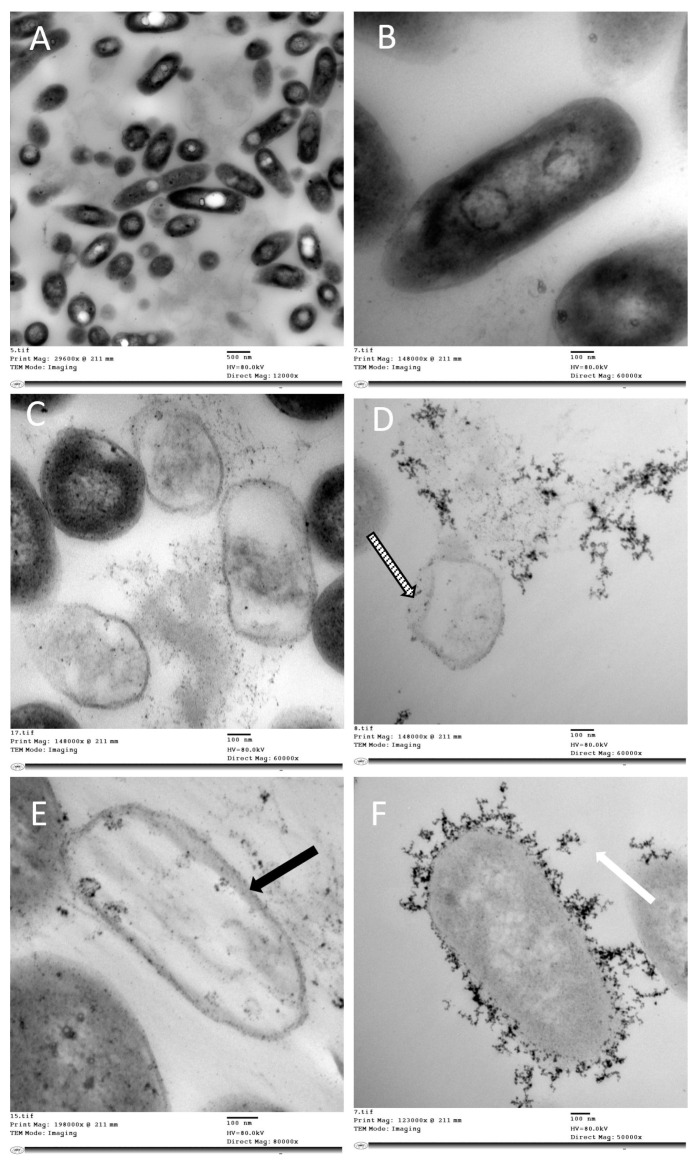
TEM images presenting the structural changes in *Acinetobacter baumannii* after treatment with rhamnolipid nano-micelles at concentrations less than the MIC. Images (**A**,**B**) show a normal cell wall outline without any itching or degradation, and with normal intracellular components. Image (**C**) is an overview of the field showing abnormal cell morphology. Images (**D**–**F**) present the possible antibacterial mechanisms of the rhamnolipid nano-micelles solution: (**D**) abnormal, degraded, itched, and permeabilized cell wall (stripped arrow); (**E**) ghost cell showing loss of intracellular components (black arrow); and (**F**) the presence of amorphous electron-dense materials (white arrow).

TEM was performed to identify the ultrastructural changes and propose the possible antibacterial mechanisms of action for nano-micelles. TEM images for bacteria treated with the rhamnolipid nano-micelle solution versus untreated bacteria are presented in Figure 4 and Figure 5. Rhamnolipid nano-micelles in solution form showed different degrees of damage to *S. aureus* and *A. baumannii*. As revealed in Figure 4, untreated samples of *S*. *aureus* showed a normal cell shape with no abnormal structural changes (Figure 4A), and the bacterial cell walls appeared intact without any itches. Normal cell division was recorded as confirmed by the normal central plane of division (Figure 4B). Upon treating *S. aureus* with the rhamnolipid nano-micelles solution, structural changes were observed (Figure 4C). These involved abnormal cell division, as confirmed by the loss of the central plane of cell division (Figure 4D, white arrow) and disruption of the cell walls (Figure 4E, striped arrow), leading to leakage of intracellular components and the formation of ghost bacterial cells (Figure 4F, black arrow).

On the other hand, TEM images of untreated *A. baumannii* showed a normal cell structure (Figure 5A). Moreover, the cell wall appeared intact without any itches or detached parts, and the intercellular components were maintained (Figure 5B). Upon treating *A. baumannii* with the rhamnolipid nano-micelles solution, TEM images showed structural changes (Figure 5C), including cell wall disruption (Figure 5D, striped arrow), leading to loss of intracellular components and the formation of ghost bacterial cells (Figure 5E, black arrow), in addition to the presence of amorphous electron-dense materials (Figure 5F, white arrow). The presence of electron-dense amorphous substances was observed in the *A. baumannii* images. Although they are unknown substances and need further investigation, similar substances were recorded in a previous study when *P. aeruginosa* was treated with rhamnolipids at a concentration of 300 µg/mL [38].

### 2.4. Cell Viability

**Figure 6:**
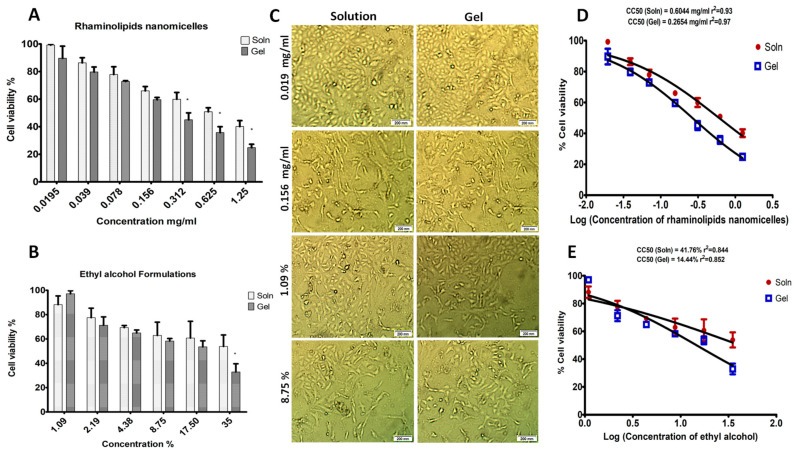
MTT cytotoxicity study of rhamnolipid nano-micelles and ethyl alcohol formulations on normal human dermal fibroblasts (HDFa): (**A**) Cell viability percentage of HDFa after treatment with different concentrations (0.0195 to 1.25 mg/mL) of rhamnolipid nano-micelle solution and gel. (**B**) Cell viability percentage of HDFa after treatment with different concentrations (1.09 to 35%) of ethyl alcohol solution and gel. Significant differences in the cytotoxic effects of rhamnolipid nano-micelles and ethyl alcohol formulations on HDFa cells are denoted with asterisks * at *p* < 0.05. (**C**) Phase-contrast microscopy images of rhamnolipid nano-micelle formulations at concentrations of 0.019 and 0.156 mg/mL, and of ethyl alcohol formulations at concentrations of 1.09% and 8.75%. Determination of the cytotoxic concentration responsible for the death of 50% of HDFa cells (CC50) for (**D**) rhamnolipid nano-micelle solution (0.625 mg/mL) and gel (0.2654 mg/mL), and (**E**) ethyl alcohol solution (41.76%) and gel (14.44%). Results are an average of three independent experiments with three replicates each.

Rhamnolipid nano-micelles and ethyl alcohol formulations were assessed for their cytotoxicity, as discussed in Section 4.2.6. As presented in Figure 6A,B, the percentage of cell viability was dependent on the nano-micelle concentration in both the solution and gel formulations, where an increase in nano-micelle concentration was accompanied by a reduction in cell viability percentage. The nano-micelle solution had a non-significant (*p* > 0.05) but higher cell viability percentage than the gel at nano-micelle concentrations ranging from 0.0195 to 0.156 mg/mL, and a significantly (*p* < 0.05) higher cell viability percentage at nano-micelles concentrations ranging from 0.312 to 1.25 mg/mL. Concerning ethyl alcohol formulations, ethyl alcohol solution had a significantly (*p* < 0.05) higher cell viability percentage than ethyl alcohol gel, but only at an ethyl alcohol concentration equivalent to 35%.

The effect of the concentration of rhamnolipid nano-micelles and ethyl alcohol on the percentage of cell viability was further confirmed by phase-contrast microscopy images (Figure 6C) for cells treated with different concentrations of rhamnolipid nano-micelles and ethyl alcohol formulations. As seen, the rhamnolipid nano-micelle solution showed higher cell viability compared to the gel formulation at the tested concentrations. By increasing the concentration from 0.0195 to 0.156 mg/mL, a reduction in cell viability was observed. The lowest concentration (1.09%) of ethyl alcohol formulations (solution and gel) showed a slightly lower cell viability percentage compared to rhamnolipid nano-micelle formulations. By increasing the ethyl alcohol concentration from 1.09 to 8.75%, a reduction in cell viability was also observed.

The cytotoxic concentration responsible for the death of 50% of cells (CC50) was calculated for each formulation of rhamnolipid nano-micelles and ethyl alcohol, and is presented in Figure 6D,E, respectively. The calculated CC50 values for the rhamnolipid nano-micelle solution, rhamnolipid nano-micelle gel, ethyl alcohol solution, and ethyl alcohol gel were 0.6044 mg/mL, 0.265 mg/mL, 41.76%, and 14.44%, respectively.

## 3. Discussion

Hospital-acquired infections (HAIs) are a major cause of morbidity and mortality, and are considered to be the second most prevalent cause of death globally [39]. Currently, many resistant pathogenic microorganisms have been identified [40], and this makes the situation worse. This could be explained by the worldwide battle against existing multidrug-resistant infectious diseases [14,41,42,43,44,45,46,47,48,49,50,51,52,53]. In the last 35 years, at least 30 novel contagious and communicable diseases have appeared [54]. Coronavirus disease 2019 (COVID-19) is one of these contagious diseases that has emerged recently, and has had a socioeconomic, environmental, and ecological impact, as well as badly affecting the mobility of the global population [1]. HAIs cause a substantial financial burden at the individual, community, and public levels [2,55]. More importantly, the burden of HAIs is estimated to be up to 18 times higher in developing countries when compared with developed countries [16,56].

However, HAIs are frequently preventable through infection prevention and control measures, with hand hygiene as a key activity [16,57]. As previously explained, alcohol-based hand sanitizers are recommended to maintain hand hygiene when water and soap are not accessible [58,59], due to their broad-spectrum antimicrobial activity, availability, and safety profiles [15]. However, their frequent administration has been reported to be accompanied by several hazards [14,15], as previously discussed. These hazards are accompanied by loss of skin cell integrity, reducing its main function as a biological barrier to protect the body against harmful pathogens, and causing a higher incidence of microbial infections [19]. Thus, finding alternative strategies for hand hygiene with less hazardous side effects is highly recommended.

The application of nanotechnology is a growing field that is interested in the production of fibers [60] and particles at nanometer scale to improve therapeutic activity and reduce the side effects of medicines, and could be also used for diagnostic purposes [52,61]. In addition, nanotechnology has also been reported as a promising strategy to combat many other viruses—including SARS-CoV-2 [14,40,61,62,63,64,65]—and multidrug-resistant bacteria [66,67,68].

Rhamnolipids are biosurfactants that are microbially produced [69], and have many unique characteristics, such as biodegradability [70], low toxicity [71], low skin irritation potential [72], and antimicrobial activity against a variety of pathogens [73,74]. Many studies [75,76,77], as well as our recent study [14], have demonstrated the antimicrobial potential of rhamnolipids against selected Gram-positive and Gram-negative bacteria, as well as their potential antiviral activity against SARS-CoV-2 [14]. However, to the best of our knowledge, no one has proposed the application of rhamnolipid nano-micelles as a potential hand sanitizer instead of alcohol-based hand sanitizers. Moreover, no studies have been performed to compare the antibacterial activity and safety profile of rhamnolipid nano-micelles versus ethyl alcohol 70%.

In the present study, rhamnolipids were produced from *P. aeruginosa* strain LeS3, and then rhamnolipid nano-micelles were prepared following our previously reported protocol [14]. Then, two pharmaceutical formulations of hand sanitizers (solution and gel) made from rhamnolipid nano-micelles and ethyl alcohol 70% were prepared.

The particle size of nano-micelles measured with the Malvern Zeta-sizer showed that the nano-micelles had a much smaller size in solution (129 nm) than in gel (263 nm) formulation. Our data are consistent with data reported in the literature, where the particle size of a nano-emulsion formulated into Carbopol 940 gel was higher than that of the original nano-emulsion [78,79]. The larger particle size recorded for the gel formulation might be attributed to the presence of polymer molecules (Carbopol 940), which might render it difficult to detect a single particle size.

TEM images obtained for rhamnolipid nano-micelle formulations showed spherical nano-micelles with no signs of aggregation, except that the nano-micelles were smaller in size than recorded by the Malvern Zeta-sizer. This difference is most likely attributable to the different techniques applied to discriminate the particle size of the nano-micelles. All samples produced were stable, as revealed from their zeta potential values (≥−30); however, the zeta potential for the gel formulation was less than that recorded for the solution. This might be attributable to the acidic nature of Carbopol 940, with the possibility to interact with the oxygen atom of rhamnolipids and, thus, reduce the negative charge of the rhamnolipid nano-micelles [80]. Importantly, samples prepared at 10 mg/mL were monodispersed, with a lower tendency to aggregate, as indicated by their PDI values (PDI ≤ 0.3). However, by diluting the samples, the PDI values for some samples were increased (PDI > 0.3), indicating a loss of sample mono-dispersity. This is consistent with our previous publication [14] and other studies reporting that PDI < 0.3 [81,82,83] is indicative of good homogeneity and is suitable for drug delivery applications.

The viscosity of the topical formulation is inversely correlated with its spreadability [84]. Thus, it is essential to determine viscosity so as to have an idea about the spreadability of the gel formulation on the skin. Therefore, the viscosity of the gel formulations of rhamnolipid nano-micelles and ethyl alcohol 70% was found to be equal to 2094 ± 19 and 2957 ± 22 cps, respectively. Although these values differ from the viscosity of the commercial alcohol-based hand sanitizer product (2344 ± 25 cps), they show a good consistency for the skin.

The measured pH of the rhamnolipid nano-micelle solution and gel was 6.32 ± 0.02 and 5.9 ± 0.2, respectively, while the pH values of the ethyl alcohol 70% solution and gel were 6.2 ± 0.19 and 5.88 ± 0.03, respectively. These figures are close to the pH of the commercial hand sanitizer solution (6 ± 0.2) and gel (5.88 ± 0.02), and they lie within the acceptable pH range (4–7) recommended for formulations applied to the skin [85,86]. Therefore, all formulations produced are suitable for application on the skin, and are unlikely to induce irritation [87,88,89]. It has been scientifically proven that the pH of a healthy skin surface is slightly acidic, with pH < 5 [90]. It is desirable to maintain this acidic pH so as to preserve skin homeostasis and the skin microbiome, as disruption of this acid mantle helps harmful bacteria to grow, and leads to adverse skin conditions such as eczema, acne, dermatitis, etc. [91,92].

The rhamnolipid nano-micelle formulations demonstrated their antibacterial activity against both tested Gram-negative and Gram-positive bacteria; however, Gram-positive bacteria were more susceptible to rhamnolipid nano-micelles. This is consistent with the literature [14,75,77,93,94], where rhamnolipids enriched with mono-rhamnolipids compared to di-rhamnolipids were reported to be more active against Gram-positive bacteria than Gram-negative bacteria [14,83]. This was attributed to the different composition of the cell walls among these bacteria [14]. The reduced effect on Gram-negative bacteria could be attributed to the presence of the lipopolysaccharide outer membrane that confers protection to the cell [77]. Similarly, de Freitas Ferreira et al. [77] reported that rhamnolipids enriched with mono-rhamnolipids compared to di-rhamnolipids revealed a resistance among Gram-negative strains.

The rhamnolipid nano-micelle solution was demonstrated to have a lower MIC value than the gel formulation for the tested organisms. This might be attributable to the lower probability of nano-micelles’ release from the viscous gel. This is consistent with a previous study where the antibacterial activity of 46 hand sanitizers was tested and it was found that viscous hand sanitizers were associated with reduced zones of inhibition, and exhibited slower killing kinetics. This was attributed to the slower release of active substances from viscous gel formulations [95].

Furthermore, a bacterial time–kill curve study was performed to determine the effective antimicrobial time for the rhamnolipid nano-micelle solution. By tracking the bacterial count at the MIC of rhamnolipid nano-micelles (Figure 3), it was observed that both bacteria maintained their initial growth inoculum (5 log cfu/mL), with no reduction in the value of Log cfu/mL when the test was run in TSB. Conversely, 100% reduction in bacterial count was recorded for *S. aureus* and *A. baumannii* when incubated in PBS for 4 and 8 h, respectively. The recorded differences in the antibacterial activity of rhamnolipid nano-micelles might be attributable to the pH of the medium used to run the time–kill curve study. The initial pH values of TSB and PBS containing the MIC of rhamnolipid nano-micelles were 7.8 and 6.3, respectively; however, TSB’s pH tends to increase slightly upon incubation with bacteria. TSB is enriched with proteins (which is not the case in PBS) and, thus, upon bacterial inoculation, alkaline byproducts are produced in the medium due to protein metabolism by bacteria, and this could explain the slight increase in pH for TSB. The alkaline pH favors the presence of ionic forms of rhamnolipids and, thus, reduces the effective interaction with bacteria, hindering the antibacterial activity of rhamnolipid nano-micelles.

These results are in agreement with a previous study [77], where the antibacterial activity of rhamnolipids at a concentration equivalent to the MIC was tested against *S. aureus* in a medium with a different pH, and it was reported that rhamnolipids showed a bactericidal effect at pH 5 and a bacteriostatic effect at pH 6. This was attributed to the presence of anionic or non-ionic forms of rhamnolipids, depending on the pH value of the medium. At pH < pKa, which is estimated to be 5.9 for mono-rhamnolipids [96], the polar groups are protonated and the non-ionic form predominates, and this favors rhamnolipid interaction with bacteria interaction and, thus, bacterial eradication.

The current findings are also consistent with a previous study that designed antimicrobial peptides (DAPs)—laboratory-synthesized antimicrobial peptides with broad-spectrum antibacterial activity, which were reported to inhibit the bacterial growth when the incubation medium was tryptic soy broth (TSB), nutrient broth (NB), Mueller–Hinton broth (MHB), or adjusted Mueller–Hinton broth (adj MHB) to a lesser extent than buffers of different pH, and this was attributed to the presence of nutrients in the culture medium, which are postulated to be metabolized, resulting in the production of alkaline byproducts [97].

TEM images of bacteria treated with a rhamnolipid nano-micelle solution indicate the possible mechanisms of antibacterial activity of the rhamnolipid nano-micelles (Figure 4 and Figure 5). Several potential mechanisms were identified, including the permeabilization of cell walls, and the releasing of intracellular components which, in turn, leads to cell death. This is consistent with the findings of previously reported studies [14,98,99], where the antibacterial activity of rhamnolipids was attributed to their solubilizing effect on the phospholipid bilayer of the bacteria, thereby increasing their permeability and the outflow of metabolites and cellular components. Such a change in phospholipid bilayer structure and function was previously reported to affect protein conformation, transport, and energy generation, ultimately leading to bacterial cell death. These proposed mechanisms of action are similar to what was reported for alcohol-based hand sanitizers to dissolve the lipid membranes of microorganisms, thereby inactivating them [100]. However, alcohol-based hand sanitizers have been reported to have adverse effects on the skin, as previously discussed [15,101].

To ascertain the potential of rhamnolipid nano-micelles as hand sanitizers to replace ethyl alcohol, it was essential to assess their cytotoxicity to dermal fibroblast cells versus ethyl alcohol (Table 2 and Figure 6). The cytotoxicity concentrations corresponding to 50% cell viability (CC50) for the rhamnolipid nano-micelle solution, rhamnolipid nano-micelle gel, ethyl alcohol solution, and ethyl alcohol gel were found to be 0.6044 mg/mL, 0.265 mg/mL, 41.76% (328.75 mg/mL), and 14.44% (113.9 mg/mL), respectively.

The recommended hand sanitizer should have broad-spectrum antimicrobial activity. Its administration should not be associated with any harmful effect on the skin, such as skin irritation, redness, dryness, or cracking. Thus, the recorded MIC values should be less than the CC50 for an antimicrobial agent to be applied safely as a hand sanitizer, so as to avoid any harmful effect(s) on skin cells. Looking at ethyl alcohol, the CC50 of the ethyl alcohol solution and gel was 41.76%, and 14.44%, respectively. These values are much less than the recommended concentration of ethyl alcohol formulations (ethyl alcohol 70%) that is approved for hand sanitation [16,17,102]. This might explain the adverse effects reported with the frequent administration of alcohol-based hand sanitizers, as previously discussed [14,15,18].

The MIC values recorded with the rhamnolipid nano-micelle gel were 0.078 and 0.625 for *S. aureus* and *A. baumannii,* respectively, corresponding to a cell viability % of 72.8 ± 0.5 and 35.7 ± 4.2, respectively. The MIC value for *A. baumannii* was 0.625 mg/mL, i.e., around three times greater than the CC50 value determined for the rhamnolipid nano-micelle gel (0.265 mg/mL) (Table 2). Therefore, the rhamnolipid nano-micelle gel has effective antibacterial activity against the tested bacteria at a concentration higher than its determined CC50; thus, its application might be associated with adverse effects on the skin.

Regarding the rhamnolipid nano-micelle solution, the MIC values recorded for *S. aureus* and *A. baumannii* were 0.039 and 0.312, respectively; these MIC values correspond to cell viability percentages equivalent to 86.5 ± 3.69% and 60 ± 4.9%, respectively. Thus, the MIC values of the rhamnolipid nano-micelle solution recorded against the tested bacteria were less than the CC50 determined for the rhamnolipid nano-micelle solution (0.6044 mg/mL) (Table 2). In other words, the solution of rhamnolipid nano-micelles has effective antibacterial activity against the tested Gram-positive and Gram-negative bacteria at a concentration lower than its CC50; therefore, it has the best safety profile among the tested formulations, i.e., its application to the skin might be associated with minimal or no harmful effects, such as those recorded with alcohol-based hand sanitizers, e.g., redness, inflammation, skin dryness, or cracking.

Our data are consistent with a previous study that reported the biocompatibility of rhamnolipids with human skin. Moreover, they were reported to have antioxidant and anti-inflammatory potential, along with surface-moisturizing properties and a very low irritability effect on human skin [103,104,105]. In addition, rhamnolipids have been reported to promote fibroblast and epithelial cell proliferation and collagen deposition on the skin [106,107]. Taken together, the rhamnolipid nano-micelle solution demonstrated the potential for effective antibacterial activity and the highest safety profile. Therefore, it might have the potential to replace alcohol-based hand sanitizers. However, must still be tested in the future among healthcare workers in hospitals to ensure its antimicrobial efficacy and safety compared to alcohol-based hand sanitizers.

## 4. Materials and Methods

### 4.1. Materials

Microbiological media (tryptone soy broth; TSB, and tryptone soy agar; TSA) were purchased from Hi-Media, India. Peptone and sodium chloride were purchased from Oxoid, UK. Hydrochloric acid, ethyl acetate, and sulfuric acid were purchased from Honeywell™, Charlotte, NC, USA. l-rhamnose was purchased from Sigma-Aldrich, Darmstadt, Germany. Orcinol was obtained from SDFCL, Chennai, India. Carbopol gel, phosphate-buffered saline (PBS) tablets, and absolute ethyl alcohol were purchased from Merck, Darmstadt, Germany. All chemicals and reagents were of analytical grade.

Two hospital-acquired human pathogenic bacteria—*Acinetobacter baumannii* and *Staphylococcus aureus*—were collected from a clinical setting in Cairo, Egypt. They were identified as multidrug-resistant bacteria.

The adult human dermal fibroblast (HDFa) cell line (PCS-201-012, HDFa) was purchased from the American Type Culture Collection (ATCC). EMEM medium, fetal bovine serum (FBS), non-essential amino acids (NEAAs), penicillin, and streptomycin were supplied by Thermo Fisher (Hessen, Germany). MTT reagent was supplied by Thermo Fisher, Germany. All other materials were purchased from Sigma-Aldrich and used as supplied.

### 4.2. Methodology

#### 4.2.1. Production of Rhamnolipids

The production of rhamnolipids was carried out following the previously reported protocol, using the shake-flask technique [14,108], and then they were further purified from the production medium by acid precipitation and organic solvent extraction [109]. Briefly, *Pseudomonas aeruginosa* strain LeS3 was grown in TSB to obtain an OD_600_ of 0.8, corresponding to a density of 8 log cfu/mL. A 250 mL Erlenmeyer flask containing 100 mL of a sterilized production medium was formulated from chicken carcass soup (CCS) containing 5% chicken fat and 0.5% NaCl. The sterilized CCS was inoculated with 1% of the overnight bacterial culture. Inoculated flasks were then incubated in an orbital shaker (Vision Scientific Co., Ltd., Bucheon, Korea; VS-8480SR) at 30 °C and 150 rpm for 5 days. At the end of the incubation period, bacterial cells were removed from the culture broth by centrifugation at 10,000 rpm and 5 °C for 10 min (Sigma-Aldrich, 3-16PK) to obtain cell-free supernatant (CFS). The CFS was acidified to pH 2.0 using 1N HCl and stored overnight at 5 °C. Rhamnolipids were then extracted using an equal volume of ethyl acetate. A yellow–brown viscous paste of rhamnolipids was obtained and then stored in the fridge until further use.

#### 4.2.2. Preparation of Antimicrobial Solutions

Rhamnolipid nano-micelles were prepared following our previously published protocol [14]. Briefly, an aqueous solution of rhamnolipids at a concentration of 10 mg/mL was sonicated in phosphate-buffered saline (PBS, 10 mM, pH 7.4) using a probe sonicator (Hielscher Ultrasonics, Berlin, Germany) to form rhamnolipid nano-micelles. Ethyl alcohol 70% (*v*/*v*) was prepared by mixing 70 mL of absolute ethyl alcohol with 30 mL of double-distilled water (Merck KGaA, Darmstadt, Germany).

#### 4.2.3. Preparation of Antimicrobial Gels

The rhamnolipid hand gel was prepared by allowing Carbopol 940 (25 mg) to swell in a mixture of glycerin (0.25 mL) and PBS (0.75 mL, 1 mM, pH 7.4) for 15 min, followed by the addition of nano-micelles dispersed in PBS buffer (12.5 mg/mL, 4 mL) to this mixture. The resultant mixture was stirred at room temperature (magnetic stirrer at 800 rpm) until a homogeneous yellowish mixture with no lumps was obtained. To adjust pH and enhance gelation, triethanolamine was added dropwise to the mixture while stirring to form the gel [110,111].

Ethyl alcohol 70% gel was prepared by allowing Carbopol 940 (0.025 gm) to swell in a mixture of glycerin (0.25 mL) and ethyl alcohol 70% (0.75 mL) for 15 min. Then, another 4 mL of ethyl alcohol 70% solution was added, and the whole mixture was stirred at room temperature (magnetic stirrer at 800 rpm) until a homogeneous, transparent, colorless mixture with no lumps was obtained. This was followed by dropwise addition of triethanolamine while stirring to form the gel.

#### 4.2.4. Characterization of Antimicrobial Formulations

##### Particle Size and Zeta Potential

The particle size and zeta potential of the rhamnolipid nano-micelle formulations were determined using a Malvern Zeta-sizer Nano ZS (Malvern Instruments Ltd., Malvern, UK) at 25 °C ± 0.1. Samples were diluted in PBS to give a count rate ranging from 50 to 300 KCPs.

##### Transmission Electron Microscopy

The rhamnolipid nanomicelle hand sanitizer formulations (solution and gel) were imaged by TEM (H-700, Hitachi Ltd., Tokyo, Japan), at an accelerated voltage of 80 kV, using the negative staining method. The rhamnolipid nanomicelle hand sanitizer formulations were diluted (1:50) with double-distilled water, and then a drop of the diluted solution was spread on a mesh copper grid coated with carbon film and kept for 5 min to dry. Then, a drop of phosphotungstic acid (2% *w*/*w*) was added to the grid for 50 s, and the excess liquid was removed using filter paper.

##### Determination of pH and Viscosity

The pH and gel viscosity of the rhamnolipid nanomicelle and ethyl alcohol 70% formulations were determined at room temperature. The pH was determined using an Ohaus Economical pH bench meter (starter 3100, New Haven, CT, USA) that was previously calibrated with three standard buffer solutions (pH of 4, 7, and 10). Gel viscosity was determined by Lamy Rheology (B-one Plus, Champagne au Mont d’Or, France) using 6 spindles at 50 rpm for 5 min. A commercially available ethanol-based hand sanitizer gel (KiviHand gel hand sanitizer, produced by KiviHand Company, Cairo, Egypt) was used as a standard for comparison [112,113,114]. The pH and viscosity of the commercial hand sanitizer gel were 5.88 ± 0.02 and 2344 ± 25 cps, respectively, while the pH of the commercial hand sanitizer solution was 6 ± 0.2.

#### 4.2.5. Antimicrobial Activity

##### Determination of Minimum Inhibitory Concentration (MIC)

The antibacterial activity of rhamnolipid nano-micelles and ethyl alcohol 70% formulations were investigated against multidrug-resistant strains of *Acinetobacter baumannii* and *Staphylococcus aureus.* MIC (the minimum concentration that inhibited visible growth) was determined only for the rhamnolipid nano-micelle formulations by microdilution assay [115]. Briefly, bacterial isolates were grown overnight in TSB at 37 °C until an OD_600_ of 0.8 was reached. Twofold serial dilutions of the rhamnolipid nano-micelle solution and gel (ranging from 0.0195 to 10 mg/mL) in TSB were carried out, and 100 µL of each concentration was added to a 96-well plate. Then, each well was inoculated with 10 µL of a final bacterial inoculum containing 5 log cfu/mL. This was followed by incubation of the plates for 18–24 h at 37 °C, and subsequent determination of MIC. The same experimental conditions were applied to assess the antimicrobial activity of the ethyl alcohol 70% formulations (solution and gel).

A set of control samples was prepared. The control for the rhamnolipid nano-micelles was a sterile broth medium containing rhamnolipid nano-micelles at each concentration, in the absence of tested bacteria. The positive control was a broth medium inoculated with the tested bacteria. The negative control was a sterile broth medium in the absence of both rhamnolipid nano-micelles and the tested bacteria. Two independent experiments were performed, where each sample was prepared in triplicate.

##### Time–Kill Curve Study of the Rhamnolipid Nano-Micelles Solution

A time–kill curve study was performed to determine the effective time of antibacterial activity of the rhamnolipid nano-micelles solution. Briefly, an overnight culture of *Staphylococcus aureus* and *Acinetobacter baumannii* adjusted to 5 log cfu/mL was inoculated with the MIC of the freshly prepared rhamnolipid nano-micelle solution (either in PBS at pH 7.4 [116] or TSB medium). The inoculum was taken at time intervals and serially diluted in dilution fluid (0.9 NaCl and 1% peptone). One hundred microliters of each dilution was then spread over the surface of well-dried TSA, followed by incubation for 18–24 h at 37 °C. Colonies were then counted, and the percentage of growth inhibition in comparison to controls was calculated. Two independent experiments were performed, where each sample was prepared in triplicate.

##### Transmission Electron Microscopy Studies

Fresh bacterial growth cultures of *Staphylococcus aureus* and *Acinetobacter baumannii* were separately grown overnight in the presence of rhamnolipid nano-micelles at a concentration less than the MIC. Bacterial cells were washed twice with PBS (1.5 mL × 2, pH 7.2) and then fixed with 2% glutaraldehyde (*v*/*v*) in PBS (1.5 mL). The samples were post-fixed with 1% OsO4 (*w*/*v*) in 5 mmol L^−1^ PBS for 1 h at room temperature, washed three times with the PBS buffer, dehydrated in graded ethanol, and then embedded in epoxy resin. Microtome sections were prepared for the specimens at approximately 500–1000 µm thickness using a Leica ultra-cut microtome (UCT microtome). These sections were then stained with toluidine blue and examined with a lens at a magnification power of 1×, and the sections were examined by camera (Leica ICC50 HD). Ultrathin sections at approximately 75–90 µm thickness were then prepared and double-stained with saturated uranyl acetate and lead citrate. At the chosen magnification, the grids were examined with a JEOL Transmission Electron Microscope (JEM-1400 TEM, JEOL-Hitachi, Tokyo, Japan). Images were captured by an AMT CCD Optronics camera with 1632 × 1632-pixel format as a side-mount configuration. This camera used a 1394 FireWire board for acquisition.

#### 4.2.6. Cytotoxicity Assay

##### Cell Culture

HDFa cells were cultured in a complete EMEM culture medium containing FBS (10% *v*/*v*), Earle’s Balanced Salt Solution, non-essential amino acids, L-glutamine (2 mM), sodium pyruvate (1 mM), sodium bicarbonate (1500 mg/L), penicillin G sodium (10.000 UI), streptomycin (10 mg), and amphotericin B (25 μg), followed by incubation of cells at 37 °C and 5% CO_2_, and the culture medium was refreshed every 24 h. As the density of cells was 70 to 90%, they were sub-cultured to achieve the desired density for the cytotoxicity test. For the sub-culture, the cell culture medium was first removed, and the flask was washed twice with PBS. To detach the cells from the culture flask, trypsin/EDTA was deposited; subsequently, this cell suspension was mixed with a fresh complete culture medium in Falcon tubes. Finally, the cells were collected by centrifuging at 1500 rpm, and then adjusted to the destiny required for the cytotoxicity test.

##### MTT Cytotoxicity Assay

HDFa cells (4.5 × 10^3^ cells/well) were seeded into 96-well culture plates. Complete culture medium (100 µL; EMEM with FBS (10% *v*/*v*), Earle’s Balanced Salt Solution, non-essential amino acids, l-glutamine (2 mM), sodium pyruvate (1 mM), sodium bicarbonate (1500 mg/L), penicillin G sodium (10.000 UI), streptomycin (10 mg) and amphotericin B (25 μg)) was added to the cells, followed by incubation for 24 h at 37 °C and 5% CO_2_. The prepared formulations (gel or solution) were double-diluted with complete culture medium to prepare a set of concentrations ranging from 0.0195 to 1.25 mg/mL for the rhamnolipid nano-micelles, and from 1.09% to 35% for ethyl alcohol. Subsequently, the complete culture medium was removed from each well, and the diluted tested samples (100 µL) were added, followed by incubation for 24 h at 37 °C and 5% CO_2_. Then, the culture medium containing the formulations was removed, and a fresh complete culture medium (100 µL) was added. MTT reagent (20 µL, 1 mg/mL) was added to each well, followed by incubation for 4 h at 37 °C and 5% CO_2_. This was followed by carefully removing the complete culture medium and adding the solubilizing agent sodium dodecyl sulfate hydrochloride salt (SDS-HCL, 100 μL) to dissolve the formazan crystals. Finally, a microplate reader (Multilabel Plate Reader, PerkinElmer, Boston, MA, USA) was used to measure the absorbance of the treated cell suspension at a wavelength of 570 nm. The percentage of cell viability was calculated by dividing the test absorbance by the control absorbance and multiplying the result by 100. A set of control samples was prepared—untreated HDFa cells as well as cells treated with blank samples (blank gel and PBS used to prepare formulations) were cultured under the same conditions.

Furthermore, phase-contrast microscopy images of HDFa cells treated and incubated for 24 h with different concentrations of rhamnolipid nano-micelles and ethyl alcohol formulations were taken to further ascertain the concentration-dependent cytotoxic effects of these formulations on the viability of cells.

#### 4.2.7. Statistics

All statistical analysis was performed using two-way ANOVA followed by Bonferroni’s multiple comparison test. Analyses were carried out using GraphPad Prism 9.0 software at a confidence level of 95%.

## 5. Conclusions

Hand hygiene is now considered to be a critical infection prevention and control measure, being able to prevent/control various hospital-acquired infections, which are a major global health challenge. Although alcohol-based hand sanitizers are widely used due to their rapid action and broad-spectrum microbicidal activity, their frequent use has been linked to several adverse effects, such as skin sensitivity, drying, and cracks, which decrease the function of skin and promote the spread of skin infections. By comparing rhamnolipid nano-micelles (solution and gel) to ethyl alcohol 70% (solution and gel)—one of the most commonly used alcohol-based hand sanitizers—we found that the rhamnolipid nano-micelles solution demonstrated antibacterial activity comparable to that of alcohol-based hand sanitizers against tested Gram-positive and Gram-negative bacteria, but with less or no hazardous effects on the skin. The antibacterial mechanisms of rhamnolipids were identified via TEM, and they were similar to the antibacterial mechanisms reported for alcohol-based hand sanitizer, involving the solubilization of the phospholipid bilayer of the bacteria, thereby increasing the permeability and outflow of metabolites and cellular components, and leading to the death of bacteria. Taken together, rhamnolipid nano-micelles could be recommended to potentially replace alcohol-based hand sanitizers, especially with the existence of an established, cost-effective, large-scale production of rhamnolipids. However, they must still be tested in the future among healthcare workers in hospitals to ascertain their antimicrobial efficacy and safety compared to alcohol-based hand sanitizers.

## Figures and Tables

**Table 1:** Characterization of rhamnolipid nano-micelles hand sanitizers after serial dilution with PBS.

Concentration (mg/mL)	Rhamnolipid Nano-Micelles Solution		Rhamnolipid Nano-Micelles Gel	
	* Particle Size (nm) ± SD (PDI)	Zeta Potential (mv) ± SD	Particle Size (nm) ± SD (PDI)	Zeta Potential (mv) ± SD
10	129 ± 4.41 (0.25)	−67.97 ± 2.56	263 ± 19.13 (0.29)	−38.03 ± 9.24
5	165 ± 0.97 (0.30)	−61.57 ± 3.23	265 ± 1.37 (0.36)	−35.13 ± 3.23
2.5	169 ± 1.81 (0.267	−78.67 ± 8.61	310 ± 9.72 (0.38)	−42.53 ± 4.39
1.25	183 ± 23.08 (0.36)	−75.43 ± 4.74	308 ± 13.37 (0.47)	−39.83 ± 0.70
0.625	206 ± 5.23 (0.35)	−66.12 ± 5.60	206 ± 8.00 (0.45)	−43.43 ± 2.55
0.313	188 ± 46.98 (0.36)	−41.57 ± 13.70	193 ± 8.74 (0.56)	−36.00 ± 1.15
0.156	166 ± 21.70 (0.46)	−61.17 ± 3.59	215 ± 13.60 (0.39)	−33.23 ± 1.04
0.078	265 ± 33.56 (0.30)	−35.23 ± 4.32	192 ± 2.94 (0.47)	−33.93 ± 3.20
0.039	204 ± 12.54 (0.25)	−45.20 ± 3.08	170 ± 1.00 (0.46)	−36.43 ± 0.70
0.0195	191 ± 22.16 (0.33)	−47.93 ± 1.81	195 ± 17.44 (0.45)	−37.77 ± 1.96

* SD: standard deviation; PDI: polydispersity index.

**Table 2:** MIC * values of rhamnolipid nano-micelle formulations recorded for *S. aureus* and *A. baumannii*, and their CC50 ** towards human dermal fibroblast cells (HDFa).

Bacteria	Rhamnolipid Nano-Micelles Solution		Rhamnolipid Nano-Micelles Gel	
	MIC (mg/mL)	Cell Viability% ± SD	MIC (mg/mL)	Cell Viability% ± SD
*S. aureus*	0.039	86.5 ± 3.69	0.078	72.8 ±0.5
*A. baumannii*	0.312	60 ± 4.9	0.625	35.7 ± 4.2

* MIC: minimum inhibitory concentration; ** CC50: cytotoxic concentrations of the rhamnolipid nanomicelle solution and gel responsible for the death of 50% of HDFa were 0.6044 and 0.265 mg/mL, respectively.

## Data Availability

All authors are happy to share all data (including Supplementary Data).

## Supplementary Materials

The following are available online at https://www.mdpi.com/article/10.3390/antibiotics11050605/s1, Table S1: Congeners composition of the rhamnolipid (Rha) mixture produced by *P. aeruginosa* strain LeS3, as analyzed by LC/ESI-MS in both positive and negative modes.
