# Supplementary material for: Rhamnolipid Nano-Micelles versus Alcohol-Based Hand Sanitizer: A Comparative Study for Antibacterial Activity against Hospital-Acquired Infections and Toxicity Concerns
Source: Antibiotics (Basel). 2022 Apr 29;11(5):605. doi: 10.3390/antibiotics11050605 (PMC9137935; doi:10.3390/antibiotics11050605)
Supplement: Supplementary file 1 [file antibiotics-11-00605-s001.zip › antibiotics-1666343-supplementary.pdf]

Supplmentrary Materials

Table S1. Congeners composition of rhamnolipids [Rha(s)] mixture produced by *P. aeruginosa* strain LeS3 as analyzed by LC/ESI-MS in both positive and negative modes.

Rha(s) congeners	m/z							
	**Mol f**	**Mol wt**	**[M-H]$^-$**	**[M+H]$^+$**	**[M+Na]$^+$**	**[M+K]$^+$**	**[M-H+Na$_2$]$^+$**	**% abundance**
**Mono-rhamnolipid (rhamnolipid 1) congeners**								
**R -C_8**	$C_{14}H_{26}O_7$	306					351	12.8
**R-$C_{8:1}$**	$C_{14}H_{24}O_7$	304			327			25.2
**R-$C_{8:2}$**	$C_{14}H_{22}O_7$	302			325			29.4
**R -$C_{9:1}$**	$C_{15}H_{26}O_7$	318			341			0.3
**R-C_{10}**	$C_{16}H_{30}O_7$	334			357		379	13.6
**R-$C_{10:2}$**	$C_{16}H_{26}O_7$	330			353			0.12
**R-C_{12}**	$C_{18}H_{34}O_7$	362			385			0.24
**R-$C_{12:2}$**	$C_{18}H_{30}O_7$	358		359	381			2.28
**R-C_{13}**	$C_{19}H_{36}O_7$	376					421	0.56
**R-$C_{13:2}$**	$C_{19}H_{32}O_7$	372			395			0.8
**R-C_{14}**	$C_{20}H_{38}O_7$	390			413			0.06
**R-C_{15}**	$C_{21}H_{40}O_7$	404				443		0.24
**R-C_8 -C_{12}, R-C_9-C_{11}, R-C_{10}-C_{10}, R-C_{12} -C_8, R-C_{11}-C_9**	$C_{26}H_{48}O_9$	504	503		527	543		6.8
**R-C_8-C_{14}, R-C_9-C_{13}, R-C_{10}-C_{12}, R-C_{11}-C_{11}**	$C_{28}H_{52}O_9$	532	531				577	0.52
**R-C_8-$C_{14:1}$, R-C_9-$C_{13:1}$, R-C_{10}-$C_{12:1}$, R-C_{11}-$C_{11:1}$, R-$C_{8:1}$-C_{14}, R-$C_{9:1}$-C_{13}, R-$C_{10:1}$-C_{12}, R-$C_{11:1}$-C_{11}**	$C_{28}H_{50}O_9$	530			553			0.6
**R-C_{11}-C_{16}, R-C_{12}-C_{15}, R-C_{13}-C_{14}**	$C_{33}H_{62}O_9$	602		603		641		0.52
**R-C_{14}-$C_{16:2}$, R-C_{15}-$C_{15:2}$, R-$C_{14:2}$-C_{16}, R-$C_{15:2}$-C_{15}**	$C_{36}H_{64}O_9$	640		641				0.8
**Di-rhamnolipid (rhamnolipid 2) congeners**								
**R-R-$C_{12:1}$**	$C_{24}H_{42}O_{11}$	506					551	0.22
**R-R-$C_{16:1}$**	$C_{28}H_{50}O_{11}$	562				601		0.62
**R-R-C_8-$C_{10:2}$, R-R-C_9-$C_{9:2,}$ R-R-$C_{8:2}$-C_{10}, R-R-$C_{9:2}$-C_9**	$C_{30}H_{50}O_{13}$	618			641	657		1.42
**R-R-C_8 -C_{12}, R-R-C_9 -C_{11}, R-R-C_{10} -C_{10}, R-R-C_{12}-C_8, R-R-C_{11} -C_9,**	$C_{32}H_{58}O_{13}$	650	649		673			1.6
**R-R-C_{16}-$C_{16:2}$, R-R-$C_{16:2}$-C_{16}**	$C_{44}H_{78}O_{13}$	815		816				0.03

Mol F, Molecular formula
Mol wt, Molecular weight
R, Rhamnose
